# Energetic optimization of an autonomous mobile socially assistive robot for autism spectrum disorder

**DOI:** 10.3389/frobt.2022.1053115

**Published:** 2023-01-26

**Authors:** Ruben Fuentes-Alvarez, Alejandro Morfin-Santana, Karlo Ibañez, Isaac Chairez, Sergio Salazar

**Affiliations:** ^1^ Tecnologico de Monterrey—School of Engineering and Science, Mexico, Mexico; ^2^ Laboratorio Franco Mexicano de Informática y Automática UMI LAFMIA 3175 CINVESTAV-CNRS, Mexico, Mexico; ^3^ Tecnologico de Monterrey, Institute of Advanced Materials for Sustainable Manufacturing, Mexico, Mexico; ^4^ Bioprocesses Department, UPIBI, Instituto Politecnico Nacional, Mexico, Mexico

**Keywords:** socially assistive robotics, autism spectrum disorder, adaptive control, skid-steered autonomous robotic platform, facial emotion recognition

## Abstract

The usage of socially assistive robots for autism therapies has increased in recent years. This novel therapeutic tool allows the specialist to keep track of the improvement in socially assistive tasks for autistic children, who hypothetically prefer object-based over human interactions. These kinds of tools also allow the collection of new information to early diagnose neurodevelopment disabilities. This work presents the integration of an output feedback adaptive controller for trajectory tracking and energetic autonomy of a mobile socially assistive robot for autism spectrum disorder under an event-driven control scheme. The proposed implementation integrates facial expression and emotion recognition algorithms to detect the emotions and identities of users (providing robustness to the algorithm since it automatically generates the missing input parameters, which allows it to complete the recognition) to detonate a set of adequate trajectories. The algorithmic implementation for the proposed socially assistive robot is presented and implemented in the Linux-based Robot Operating System. It is considered that the optimization of energetic consumption of the proposal is the main contribution of this work, as it will allow therapists to extend and adapt sessions with autistic children. The experiment that validates the energetic optimization of the proposed integration of an event-driven control scheme is presented.

## 1 Introduction

Autism spectrum disorder (ASD) is a lifelong, non-progressive neurological condition related to brain development Volkmar et al. (2005). The classic form of autism involves a triad of impairments: social interaction, verbal communication, and non-verbal communication. These three conditions impact the perception of the environment and the way autistic persons socialize with other human beings. The World Health Organization (WHO) (2019) established that ASDs are a diverse group of conditions that are characterized by some degree of difficulty in communication, social interaction, restrictive behaviors, and sensory sensitivities that impact how they interact with and perceive society, especially in cultures where contact and social interaction are primordial as in Latin culture. Other symptoms often present in people with ASD are epilepsy, depression, anxiety, and attention-deficit hyperactivity disorder Mayo Foundation for Medical Education and Research (2017).

It is estimated that one in 160 children around the world are on the autism spectrum (Elsabbagh et al., 2012; WHO, 2019). A survey carried out by the Center for Disease Control and Prevention reports that the prevalence of ASD in 2014 was estimated to be close to one in 59 children at the age of 8 years in the United States Baio et al. (2018); CDC (2019). Prevalence among the Hispanic population of the United States ranges from 2.7 to 7.9 per 1,000 Pedersen et al. (2012). Unfortunately, the real incidence of ASD in countries such as Mexico is largely unknown; official estimates range from 1 to 4 per 1,000 inhabitants. Those estimates also show a prevalence of 40,000 affected children. The only registry available from the Mexican Health Department includes about 250 cases starting in 1980. It also recognizes that the incidence has been growing about 10–17% per year Oro et al. (2014); Tuman et al. (2008). It has been noticed that several factors such as gender tend to camouflage symptoms, generating a misdiagnosis and in some cases, a late diagnosis Schuck et al. (2019). To this day, there is no cure for autism. However, an early diagnosis and treatment can make an essential difference in the lives of many children, where three kinds of “traditional” therapies stand out: behavior and communication therapies Mcgee et al. (2000), educational therapies Bartak and Rutter (1973), and family therapies Lovaas (1987), where the patients are trained to respond to environmental changes and understand feelings and conversations, helping them react appropriately to social stimulus.

Other therapeutic methods such as animal-assisted therapies Rodríguez-Martínez et al. (2021) and socially assistive robotics (SAR) Puglisi et al. (2022) interaction have come to the scene recently as reliable therapeutic options. In particular, research on SAR has increased over the last few years. One of the main reasons for this research progress in SAR is its aim of interacting with people through social or physical assistance to deliver therapy or service Martinez-Martin et al. (2020). The exciting improvement in the user (especially in children) is because their attention is drawn through visual and mobility-based interactions which guarantee their social skill improvement Feil-Seifer and Matarić (2009). Moreover, SAR focuses on creating close and effective human–robot interaction (HRI) to quantify the progress made in a certain activity (e.g., learning, rehabilitation, and companionship) Fong et al. (2003). For effective interaction processes, SARs must have components to enable the promotion and accomplishment of embodiment, emotion, dialogue, personality, human-oriented perception, user modeling, socially simulated learning, and intention properties. The incorporation of machine learning algorithms such as HRI solutions to SAR has provided feasible interaction models during the therapies Martinez-Martin et al. (2020). On the other hand, some studies have demonstrated that autistic individuals tend to feel more comfortable when interacting with robots because they are more predictable than specialists and the environment of therapies Costa et al. (2017). Also, autistic people typically show better response to feedback provided by non-humanoid elements than humans. In addition, the non-humanoid SAR may present hypothetical advantages over humanoid robots, since they prefer objects over social environments. Therefore, the usage of SARs as therapeutic tools is considered to improve and enhance efficiency during therapy, providing more consistent outcomes.

The SAR is used as a therapeutic tool for autistic persons, which requires the accomplishment of different requirements to be effective. First , the robotic system needs to be capable of adapting its spatial position when the emotional state of the interacting user changes. Consequently, the SAR requires an intelligent method based on facial recognition techniques to identify when emotional changes occur during a therapy session. Both requirements need to be integrated through an algorithmic method, capable of broadcasting recognized emotions and mapping them into trajectories that will be realized by the SAR to modify certain emotional stages of the user. The aforementioned requirements also need to consider the energetic consumption of SARs when implementing both artificial intelligence and control algorithms for an HRI method. The optimization of its energetic autonomy will allow longer therapeutic sessions while conducting and adapting the treatment depending on the environment and the patient’s responses.

This work proposes the integration of an output feedback adaptive controller (OFAC) based on sequential super-twisting differentiators capable of optimizing the energy consumption during the trajectory tracking problem when used on an event-driven control scheme whose states will be chosen through a facial emotion recognition (FER) algorithm. The complete intelligent system is allocated to a 4-wheeled drive (4WD) as a skid-steered autonomous robotic platform (SSARP) capable of interacting with autistic children.

## 2 Materials and methods

### 2.1 Conceptualization

An experimental report generated by the Adaptive Systems Research Group from the University of Hertfordshire in the UK Dautenhahn et al. (2003) concluded that the adaptability of the interaction needs to be centered on the individual characteristics of toddlers. This statement opens the possibility of proposing the implementation of event-driven control schemes in non-humanoid robotic platforms specializing in the treatment of people on the autism spectrum. Furthermore, the integration of a facial emotion recognition system that tracks the most effective trajectories for the interaction between the robotic agent and the user can be implemented by an event-driven control strategy.

Generally, event-driven control models are driven by externally generated events. These events are usually signals taking a range of values. The distinction between an event and a simple input detonating the control is that the event is outside the control of the process that handles that event. In this way, it is well known that neural networks (NNs) are ruled by their own event production systems. More specifically, a condition becoming true causes an action to be triggered and active objects where changing the value of an object’s attribute triggers some actions.

**FIGURE 1:**
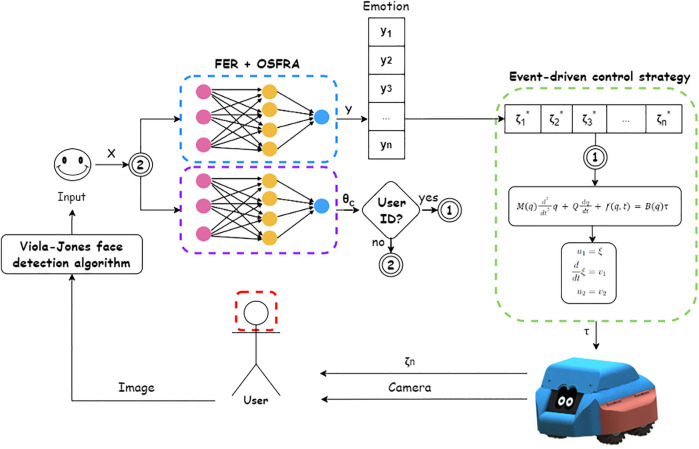
Scheme of the proposed broadcast event-driven control strategy.

The event-driven broadcast model’s purpose is to transmit an event to all the components that are integrated with the system. The main limitation of this scheme is that each of the components needs to be prepared to handle that event so that the system can respond to it. The advantage of the broadcast method is that classes of events can be integrated by registering their events with the event handler. Also, it is considered that broadcast models are more effective when integrating components distributed across different elements from a network. Finally, a broadcast model capable of registering events such as facial recognition and FER to prompt a set of trajectories to be carried by the SAR when these events occur can be proposed. The broadcast event-driven control model implemented in this work is described in Figure 1, which also depicts the whole algorithmic integration of the robotic system.

The scheme presented in Figure 1 proposes a human-in-the-loop concept, where the system depends on both the user’s identity and emotion to trigger a specific event. A one-shot facial recognition algorithm (OSFRA) and an FER algorithm handle the outputs that will serve as the events used to select a specific trajectory ζ* from a specific set of trajectories. This trajectory will define the adaptive gain selection for an OFAC, so the trajectory can be accomplished when the control signals are transmitted to the SAR motors, consuming less energy when compared to a state feedback controller (SFC). Also, it is expected that each of the trajectories affects the emotion of the autistic user, closing the loop every time the trajectory is accomplished by the SAR.

**FIGURE 2:**
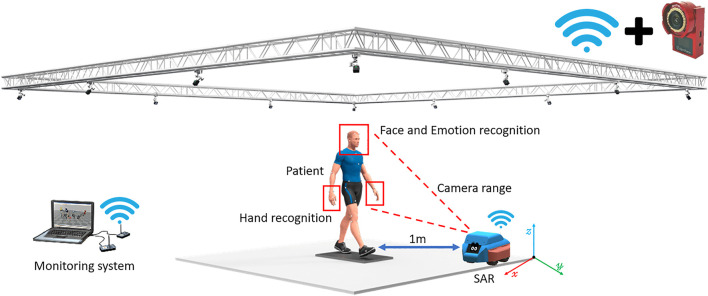
Conceptualization of the proposed SAR system. Notice that to avoid the transmission of trash data from the motion-capture system, a minimum of 12 different-sized markers need to be allocated over the surface of both the subject and the SAR by following an asymmetric pattern. This will allow solving three problems: **(A)** identifying the SAR rear, front, right, and left positions; **(B)** losing sight of the SAR; and **(C)** keeping track of the HRI.

It must be considered that the implementation proposal requires an onboard element capable of acquiring facial images from the users and a motion-capture video acquisition system to determine the *x*−*y* coordinate of the SAR (Figure 2). The robot position will feed the control algorithm through a local area network Wi-Fi connection. The network allocates the motion-capture monitoring system and the SAR connected through the Robot Operating System (ROS) running on a low-level controlling board that will manage to calculate the adaptive gains of the OFAC to transmit them to the motors and broadcast the activation states that provide the OFAC with desired trajectories ζ*.

### 2.2 Instrumentation of a skid-steered autonomous robotic platform

**FIGURE 3:**
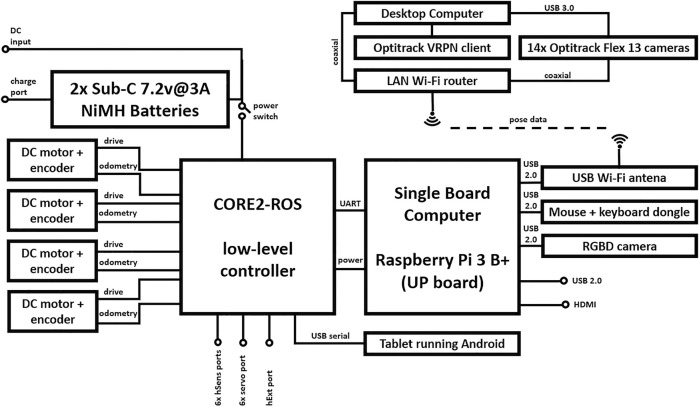
Graphic representation of the components integrating the proposed robotic platform and the connections between them.

The proposal was implemented on an SSARP following the proposal from Fuentes-Alvarez et al. (2022) and instrumented onto a 4WD Dagu Electronics chassis to be used as the SAR structural base. In particular, this robotic structure is designed to accomplish all-terrain tasks; its complex outdoor capabilities may allow this SSARP to be a potentially accurate chassis for a wheeled mobile SAR. It is worth mentioning that Dagu’s chassis complies with the amending Directive 2015/863/EU known as (RoHS 3) European Parliament and Council of the European Union (2015) and the 2011/65/EU directive European Parliament Council of the European Union (2011) on the restriction of the usage of certain hazardous substances in electrical and electronic equipment. This ensures an electrical security level when thinking of an interactive robot. Also, the actuators selected to enable the 4WD SSARP movement were Pololu metal gearmotors with an integrated 48 counts per revolution quadrature encoder, whose gear ratio is 34:1. These motors work at 6 V, providing 170 rpm over the output shaft at no load speed, and a stall current of 2.4 A when the stall torque is 3.5 kg-cm. At the end of each actuator, 120-mm spiked tires and a flexible suspension system are allocated to ensure each wheel maintains contact with the ground. A Husarion CORE2 low-level controlling board will serve as a bridge for transferring the pulse-width-modulated (PWM) signals to the motors. These signals are generated from the control algorithm, which is hosted on Raspberry Pi 3 B+ running Ubuntu and ROS. Both electronic boards are interconnected to calculate the adaptive gains through the methods established by Fuentes-Alvarez et al. (2022), which will then control the velocity of the SSARP wheels. Additionally, the Husarion CORE2 allows the connection of a tablet allocated in the frontal part of the SSARP, enabling the integration of an interactive sequence through ROS. Also, taking advantage of the tablet’s frontal camera as the OSFRA inputs, these algorithms will allow the system to deploy an event-driven control scheme described in the previous section. The aforementioned elements are put all together and connected as depicted in Figure 3. It is worth mentioning that the system does not count with a Hall effect sensor. In addition, the quantification of energy is based on the control signal and the well-known mathematical model of a DC motor.

### 2.3 Control strategy for a skid-steered autonomous robotic platform

The design of the interaction between the robotic platform and children with ASD presents a complex task. It is essential to determine how movements will ensure positive stimuli for the users while reducing the energy consumption of the SSARP-based SAR. Since there is no evidence of an adequate set of trajectories or trends for this task to be accomplished, the implemented control strategy requires the capability of reproducing a wide range of trajectories. The implemented OFAC aims to provide robustness to the SSARP trajectory tracking; these dynamic models can be described as presented in Eq. 1 when following the linearization proposed by Caracciolo et al. (1999):

$$\dddot{\zeta }=\alpha \left(q,\eta \right)v+\beta \left(q,\eta \right)+\tilde{f_{r}},$$ (1)

where *α* is a well-defined non-singular decoupling matrix considering the pseudo-velocities *η* ≠ 0, *β* contains the non-holonomic restrictions presented by the model, and *v* is a virtual control extension. Notably, the input control signal follows a certain relationship between the original state vector $q^{⊤}=\left[X,Y,\omega \right]$ obtained as:

$$v=\alpha^{-1}\left(q,\eta \right)\left[r-\beta \left(\zeta ,\eta \right)\right].$$ (2)

The term *r* refers to the jerk effect, the dynamic form of which corresponds to:

$$\dddot{\zeta }=r.$$ (3)

After integrating the jerk reference from Eq. (3), the new dynamic system of the SSARP can be rewritten as:

$$\begin{array}{c} \dot{Z}=AZ+Br\\ Z={\left[\zeta^{⊤},{\dot{\zeta }}^{⊤},{\ddot{\zeta }}^{⊤}\right]}^{⊤}, \end{array}$$ (4)

where matrices *A* and *B* are the controllable companion pair of adequate dimensions for a multiple-input and multiple-output dynamic systems. The complete mathematical process and the sequential super-twisting differentiator design to estimate $\dddot{\zeta }$ can be consulted in the study by Martínez-Fonseca et al. (2014).

After recovering the required states of *Z*, it is possible to describe the OFAC. Considering the reference path is differentiable at least twice, then there is a state variable representation given by $Z^{*}inR^{6}$ such that:

$${\dot{Z}}^{*}\left(t\right)=AZ^{*}+Bh\left(Z^{*}\left(t\right),t\right).$$ (5)

Therefore, the definition of the tracking error function *Δ* = *Z*−Z* leads to the dynamics associated with *Δ*:

$$\dot{\Delta }\left(t\right)=A\Delta \left(t\right)+B\left(r\left(t\right)-h\left(Z^{*}\left(t\right),t\right)\right),$$ (6)

and the new control input *r* corresponds to the following definition:

$$r\left(t\right)=h\left(Z^{*}\left(t\right),t\right)+K^{⊤}\left(t\right)\Delta \left(t\right),$$ (7)

where *K* satisfies

$$\dot{K}\left(t\right)=-\alpha_{Q}P\Delta^{⊤}\left(t\right)\Delta \left(t\right)-\tilde{K}\left(t\right),$$ (8)

where $\alpha_{Q}=\left\{\lambda_{\min}P^{-1/2}QP^{-1/2}\right\}$ is a positive scalar and $P\in R^{6\times 6}$ and $q\in R^{6\times 6}$ are positive definite matrices which regulate the time variation of the controller gain. Here, $\tilde{K}=K-K^{*}$ ; K* represents any possible matrix such that *A*_*K*_ = *A*−BK* is a Hurwitz matrix.

It is worth mentioning that the stability proof presented by Fuentes-Alvarez et al. (2022) ensures the trajectory tracking error convergence to a small region. Consequently, the control input signal can be reduced, allowing the optimization of the energy consumption Carabin et al. (2017) of the OFAC. Finally, the OFAC algorithm presented in this section is implemented onto the SAR throughout the ROS frame in the node named *ctrl*_*node*.

### 2.4 Face recognition and convolutional neural network for emotion recognition

Creating a link between the robot and the human being is essential for the designs of social assistance robots. The robot has to interact with humans and understand both verbal and non-verbal behaviors. Nowadays, cameras make it possible to capture the user’s movements and gestures on video so that the robot can interact with certain behavior and adapt to the changes that may occur during human–robot interaction Shen et al. (2021). This section presents an algorithmic implementation to determine the facial emotion and identity of autistic children interacting with the SAR. This implementation relevance relies on the consideration that ASD is a psychological disorder that alters the processing of emotions and, consequently, lowers the ability for social interaction and communication.

#### 2.4.1 Face tracking

The technique known as the Viola–Jones technique was used for face tracking and detection Viola and Jones (2001). The algorithm detects edge or line features and extracts them from a series of positive images that contain faces. Here, the integral image at location *x*, *y* encompasses the sum of the pixels under and to the left of *x*, *y*, as follows:

$$ii\left(x,y\right)=\underset{x^{\prime }\leq x,y\leq y^{\prime }}{\sum }i\left(x^{\prime },y^{\prime }\right),$$ (9)

where $ii\left(x,y\right)$ is the mathematical representation of the integral image, and $i\left(x,y\right)$ is the original image.

Afterward, Ockham’s razor principle is followed to implement a weak learning algorithm (AdaBoost) which picks out the single rectangle feature that best isolates the positive from the negative examples. Despite several NN algorithms that can achieve this task in a better way, this face-tracking algorithm is used because it implicitly provides a feature space subset from the main image dataset, but also requires a minimum quantity of computational resources to be deployed. Therefore, the implementation of the Viola–Jones technique, a face recognition algorithm, must be integrated after resolving the face tracking and face feature extraction.

### 2.5 Facial recognition

An OSFRA based on the generative one-shot face recognition model from Ding et al. (2018) is proposed. It is possible to implement an OSFRA algorithm once data are allocated on the feature space. Furthermore, this allows the processing unit to reduce computational time since the amount of data is decreased. Since the face-tracking strategy will obtain a one-time face sample from the first interaction with the child, the integration of a data set before the interaction with the robot is not necessary. Usually, recognition models are trained to label training data with two sets without identity overlap, where the base set {*X*_*b*_, *Y*_*b*_} contains *c*_*b*_ classes and the one-shot set {*X*_*n*_, *Y*_*n*_} contains *c*_*n*_ classes. The goal is to build a general *c*-class recognizer *c* = *c*_*b*_ + *c*_*n*_. The proposed algorithm includes two phases. The first phase comprehends a representation learning stage. The representation learning stage, where the face representation is modeled using training images from a base set, was presented in the previous section. The second phase is one-shot learning. The following stage is trained as a multi-class classifier to recognize the person in both the base set and the one-shot set based on the representation model learned in the previous stage. Hence, it is essential to balance these two sets to achieve a more effective feature representation; results obtained by Ding et al. (2018) were followed. Features extracted from the last pooling layer are used as the face representation. Certainly, the aforementioned implementation presented an over-fitting phenomenon over the new *c* recognizer, which is associated with the *c*_*k*_ distance between classes, reducing the norms of both parameters contained in the *c*_*n*_ set. To solve this problem, a center-loss method for the parameter update is proposed as in Algorithm 1 by following the results from Wen et al. (2016).

Algorithm 1 Discriminant feature learning algorithm based on center-loss functions.

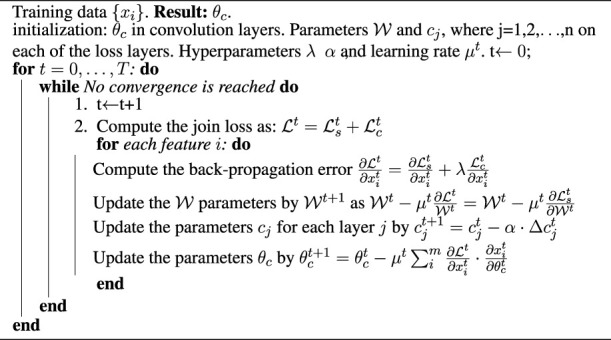

#### 2.5.1 Facial emotion recognition

The FER algorithm is based on a CNN which proposes a structure that comprises nine fully connected layers, containing four residual depth-separable convolutions. This is followed by a batch normalization operation and the implementation of the rectifier linear unit activation function. Finally, a batch normalization layer is presented to reduce covariance during the training process. The input of the CNN are the features obtained from the Viola–Jones algorithm described at the beginning of this section. For the training and validation processes of the FER algorithm, the FER-2013 dataset Zahara et al. (2020) was used. These datasets contain 460,723 and 28,709 RGB images. Also, the classes contained in the datasets are *Neutral*, *Happy*, *Surprised*, *Angry*, *Sad*, *Fear*, *and Disgust*. The output of CNN “emotion recognition” determines the movements of the mobile robot, which has predetermined control trajectories associated with each emotion.

## 3 Results

### 3.1 Trajectory tracking of a squared trajectory

**FIGURE 4:**
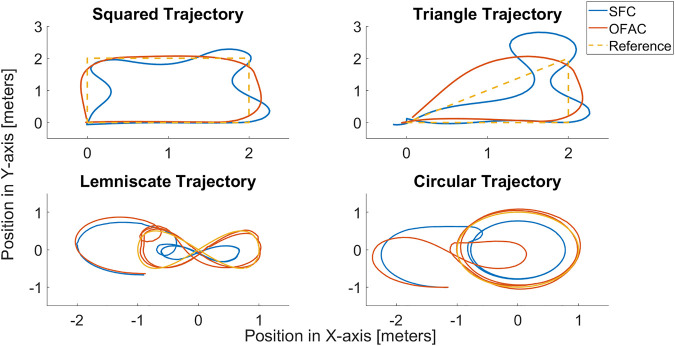
SSARP trajectory tracking of different trajectories in the X–Y plane.

Once the OFAC design is defined, it is necessary to define an adequate trajectory presenting a non-linearity effect and whose accomplishment requires a considerable amount of energy. For this example, ζ* is proposed as a 2*m* × 2*m* squared trajectory. The results obtained from the implementation of the trajectory tracking are presented in Figure 4.

It can be observed in Figure 4 that the skidding effect is present when tracking ζ*. Also, the SSARP smothers the sharp angles of the squared trajectory when turning. The accumulated error of the OFAC is reported to be .2146, while the reported SFC is .6326. This shows that the SFC can be $\tilde{3}$ times less accurate than the OFAC when following this type of trajectory. Other trajectories such as lemniscate, circular, and triangular trajectories were also tested. Results for each trajectory are presented in the following sections of this paper.

### 3.2 Absolute power consumption

**FIGURE 5:**
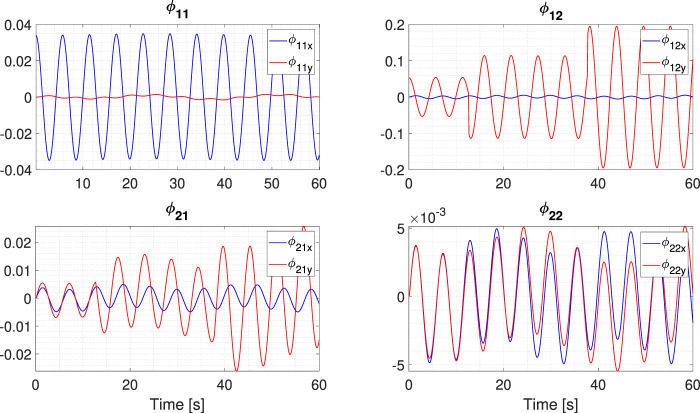
Evolution of the time-dependent terms for the observer based on the nested super-twisting algorithm.

The energy consumption of the OFAC proved to be less than that consumed by the SFC. The accumulated energy of the OFAC was 6.7255 × 10^4^ J, while the SFC accumulated consumption was 1.8351 × 10^5^ J. These results present the optimization of the absolute power consumption. Notably, the maximum amount of power required by the OFAC to track a squared trajectory is $\tilde{4}.5$ W. When traduced to the total current consumed by the DC motors at the highest energy peak, the consumption is equivalent to 0.3 A; considering the instrumentation, energy input is 14.2 VDC at 3 A, and the total load of the SAR is 5 kg. Also, the evolution of the *ϕ*_*i*,*k*_ gains implemented in the super-twisting gains through time is presented in Figure 5, showing that the amount of power consumed by the sequential super-twisting differentiator is nearly negligible. The evolution of these functions and their convergence to a zone around the origin confirms that the reconstruction of the time derivative for variable *ζ* is completed successfully in finite time. Energetic consumption results from the SSARP when following different trajectories are presented in Table 1.

**TABLE 1:** Energy consumed by the SSARP when following different trajectories in the x–y plane.

Controller	Trajectory	Energy consumption (J)
State feedback controller	Triangle	124.37
Output feedback adaptive controller	Triangle	115.88
State feedback controller	Square	141.81
Output feedback adaptive controller	Square	132.92
State feedback controller	Lemniscate	186.49
Output feedback adaptive controller	Lemniscate	183.32
State feedback controller	Circular	167.13
Output feedback adaptive controller	Circular	152.89

### 3.3 Face and emotion detection

**FIGURE 6:**
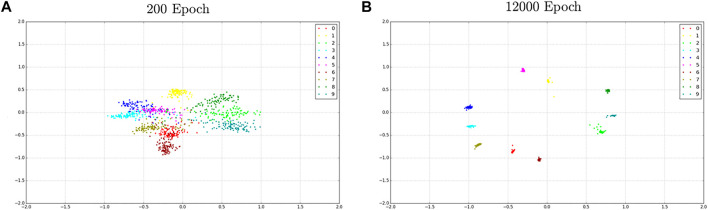
Distribution of the learned features under the joint supervision of the center-loss algorithm. Each color represents a total of nine different features from particular classes associated with a different identity. **(A)** shows the learning process after 200 epochs, while **(B)** depicts the learning process after 12,000 epochs. Notably, distances are normalized and each color represents a different identity.

The implemented OSFRA serves as an information access layer that ensures data acquisition during each patient’s therapy. The algorithm’s most remarkable strength lies in its generative capability, which means that even with a limited quantity of input parameters, it is capable of completing the recognition task by auto-generating its own feature space. Its implementation aims to give personalized follow-up to patients. Figure 6 shows the results of the OSFRA implementation running on a Linux Ubuntu distribution mounted on Raspberry Pi 3 B+.

**FIGURE 7:**
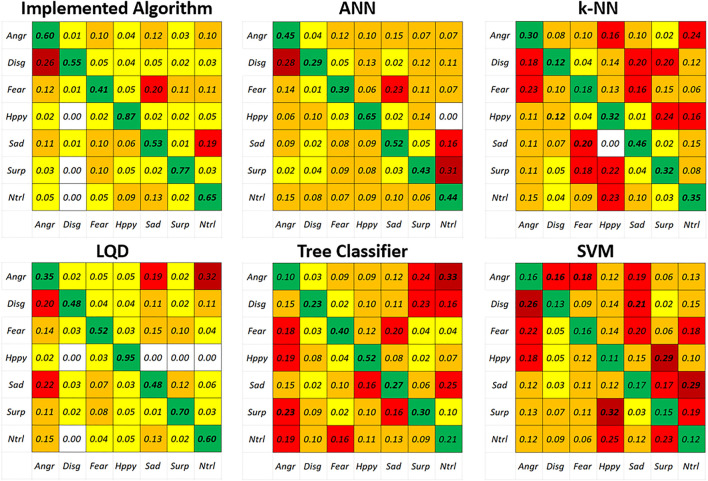
Comparison of different classification algorithms for the FER task running over the FER-2013 dataset.

The FER was implemented using the video references obtained from the onboard tablet camera. Since there is a close similarity in how children with Asperger’s syndrome and autism express their emotions and their facial expressions, tests were carried out with the help of a 16-year-old man diagnosed with Asperger’s syndrome. It is important to mention that the results obtained from the implementation of the FER algorithm provide the system with the capability of recognizing six different facial emotions: *Neutral*, *Happy*, *Surprise*, *Angry*, *Sad*, and *Fear*. Also, Figure 7 shows the true performances of the proposed classification through the comparison of the confusion matrices during the validation process against algorithms such as artificial neural networks (ANNs), k-nearest neighbors (k-NN), linear quadratic discriminator (LQD), tree classifier, and support vector machine (SVM). It must be clarified that each of the aforementioned algorithms were tested using MATLAB toolboxes, tuning the hyperparameters to obtain the best possible performance. The results obtained from k-NN, tree classifier, and SVM methods resulted in the observation of several classes being misclassified, i.e., *Sad* emotion being classified as *Fear* 20% of the times for k-NN or *Surprise* as *Happiness* 32% of the times when running SVM. After running these tests, it was decided that the *Disgusting* emotion needed to be removed from the image-set, since this class presented a higher misclassification accumulative rate when compared to others. Furthermore, the *Disgusting* expression is not considered a “valid” emotion by therapists attending to ASD children. In addition, this comparison serves as evidence of the accuracy of the implemented algorithm compared to other classification methods.

### 3.4 Prototype integration

**FIGURE 8:**
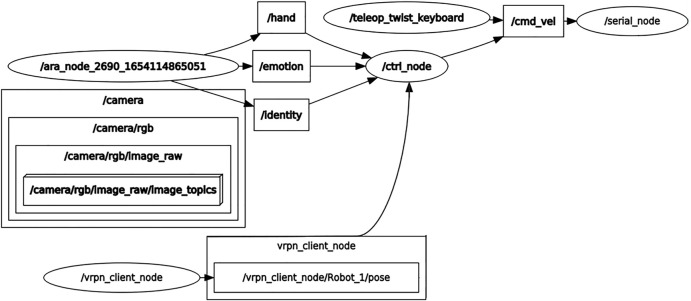
Integrated system presented as an ROS graph. This structure shows graphically how the SAR system is integrated. The main nodes *ara*_*node* and *ctrl*_*node* represent the principal energetic consumption elements of the system.

**FIGURE 9:**
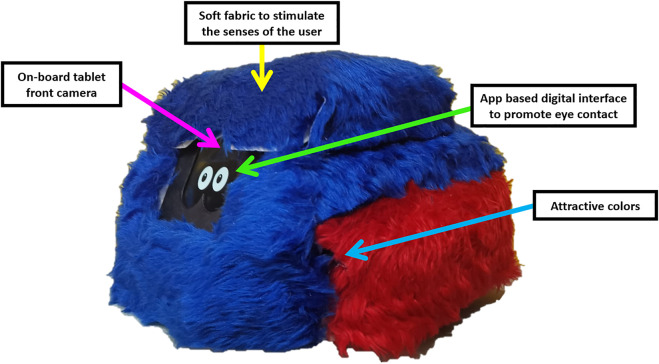
Final aspect of the proposed SAR platform. A bi-tone soft fabric cover was used to invite ASD users to establish physical contact with the SAR. *Red* and *blue* colors were selected for the cover. Also, an eye animation is projected on the onboard tablet screen, which serves as an indicator that all systems are running correctly. After its assembly process, the prototype of the SAR proposed in this work received the name of Autonomous Robot for Autism (AR4A).

The reason for proposing an event-driven control strategy, which uses externally generated events such as emotions to trigger certain control states, is centered on the contactless HRI required to avoid harmful events during therapies. This triggering role will be played by the output of both FER and the OSFRA. The connection between the OFAC, FER, and the OSFRA is carried out through the ROS *vrpn_client_node*. Infrared cameras will feed the designed ROS OFAC node (*ctrl*_*node*) with the pose of the SAR and the subject in the room’s free space through a closed local area network Wi-Fi connection. It is worth mentioning that both OSFRA and FER run in the *ara*_*node*, broadcasting the activation states that provide the OFAC with desired trajectories ζ*. Figure 8 demonstrates the algorithmic interaction during the ROS implementation, showing the involved nodes and topics for the event-driven control scheme. Finally, the proposed robotic platform AR4A (Autonomous Robot for Autism) is presented in Figure 9. A selection of red and blue plush fabric was used to cover the external structure in compliance with the autism color theory to positively stimulate the sensorial perception of children with ASD. The total height of AR4A from the ground to the top is 275.2 mm, the width from the left wheel to the right wheel is 373.5 mm, the length from front to rear is 368.68 mm, and AR4As’ total weight is $\tilde{5}$ kg.

The AR4A node implementing FER, OSFRA, and OFAC algorithms on a 4WD SSARP can be consulted on https://github.com/jorufua/AR4A.

## 4 Discussion

Persons with ASD usually develop movement-planning capacity after interacting with robotic agents. This process happens after the observation of congruent moves. Some reports conclude that the adaptability of the interaction needs to be centered on the individual characteristics of this type of patient to be more effective over time. For these reasons, it is essential to provide SARs with energy-effective control and HRI algorithms. In this work, the integration of FER + OSFRA algorithms used as HRI was proposed to denote the trajectory tracking of an SSARP-based SAR through an event-driven control scheme.

The implemented OFAC reached a mean-square error (MSE) of .2146 when compared against an SFC during a squared trajectory tracking, in which the SFC performance was .6326. Also, the OFAC presented a skidding effect when the trajectory edges were presented. These sharp turns represent the highest energy requirements for the controller. The total energy consumption during the tracking of the squared trajectory for the OFAC was 39.04 J, while the SFC consumed 100.6 J. Table 2 presents the energetic performance of both controllers.

**TABLE 2:** Comparison of the implemented OFAC against an SFC for various trajectory tracking on an SSARP-based SAR.

Trajectory	Controller	TMSE	Energy consumption (J)
Triangle	State feedback controller	.693	124.37
Triangle	Output feedback adaptive controller	.625	115.8
Square	State feedback controller	.632	141.8
Square	Output feedback adaptive controller	.214	132.9
Lemniscate	State feedback controller	.622	186.49
Lemniscate	Output feedback adaptive controller	.572	183.32
Circular	State feedback controller	.583	167.13
Circular	Output feedback adaptive controller	.552	152.82

The implemented FER algorithm reached an accuracy of 63.6% when compared against ANN, k-NN, LQD, tree classifier, and SVM for the FER classification task. It must be added that all these algorithms were tested and compared for the FER-2013 database. Table 3 presents the classification performances shown by each of the implementations after running a 5-fold cross-validation process.

**TABLE 3:** Comparison of the implemented FER against other classifiers.

Classification algorithm	5-fold cross-validation
min-Xception Arriaga et al. (2017);	65%–67%
Gogate et al. (2020)	
Proposed CNN	63.6%
ANN	45.47%
k-NN	29.28%
LQD	58.33%
Tree classifier	29.04%
SVM	14.28%

After analyzing the results obtained from comparing different FER algorithmic solutions, it can be concluded that the proposed CNN solution is slightly below the classification performance compared to the min-Xception algorithm reported in the literature. However, a deeper comparison must be carried out to determine which solution results in more efficient running on an onboard device. As a claim over this specific point, it must be said that the proposed CNN has already been implemented on the onboard Raspberry Pi device, resulting in a delay of the FER of nearly .85 s when running all the detection modules proposed in this work. When carrying out an analysis of the computational complexity of the proposed algorithms under an event-driven scheme, it is shown that the complete implementation runs on $O\left(knd^{2}\right)$ , where O(k⋅d) represents the total *k* dot products in the weight matrix *W* of the CNN and finally, at the layer level, the filtering application over the input *n*−*k*+1 times, where *n* is the length of the input. In terms of consumption, the total amount consumed by the implemented system was 34.08 W per hour.

### 4.1 Future work

Once the SAR control scheme and the recognition algorithms are proved to be functional with an acceptable energetic consumption amount, autonomous navigation based on simultaneous localization and mapping (SLAM) algorithms must be implemented. This implementation requires the modification of the instrumentation stage by adding a stereoscopic camera and a light detection and ranging device. This implementation will enable the device to not only gain navigation autonomy but also to interact in a more efficient way with patients. It must be considered that interaction times will be affected and that the implementation of other algorithms to analyze the biomechanics of the interaction during sessions must be implemented to maintain some of the advantages provided by the current system.

## Data Availability

Publicly available datasets were analyzed in this study. These data can be found here: https://www.kaggle.com/datasets/msambare/fer2013.
